# The Role of Microbiology Testing in Controlling Infection and Promoting Antimicrobial Stewardship

**DOI:** 10.1007/978-3-030-62662-4_5

**Published:** 2020-11-24

**Authors:** Louise Ackers, Gavin Ackers-Johnson, Joanne Welsh, Daniel Kibombo, Samuel Opio

**Affiliations:** 6 Global Social Justice, University of Salford, Salford, UK; 7 University of Salford, Salford, UK; 8 Infectious Disease Institute, Kampala, Uganda; 9 Pharmaceutical Society of Uganda, Kampala, Uganda

**Keywords:** Antibiotic resistance, Culture and sensitivity testing, Isolates, Rational prescribing, Empirical therapy, Antibiogram

## Abstract

This chapter discusses the Role of Microbiology Testing in Controlling Infection and Promoting Antimicrobial Stewardship. It details the role that culture and sensitivity testing has played in creating the ‘objective’ evidence base that facilitated and nurtured midwifery empowerment, task shifting and multi-disciplinary team working. The chapter presents findings of resistance patterns of isolates from women with suspected sepsis.

Objective 3.2.1 of the Uganda National Action Plan (Fig. 10.1007/978-3-030-62662-4_4) outlined key components of IPC. All the familiar ‘good housekeeping’ aspects of IPC are listed. Sub-Objective 6 encouraging ‘timely diagnosis and treatment of drug-resistant microorganisms’ stands out as a less usual element of IPC. It expresses the ‘control’ dimension of IPC and forms an essential interface with the stewardship aspects of the National Action Plan demonstrating the need for holistic approaches to AMR. Objective 3.3 of the Action Plan combines a concern to ‘Optimize Access to Effective Antimicrobial Medicines’ with access to ‘Diagnostics in Human Health’. This latter objective overlaps with Sub-Objective 6 (above) and is focused on (1) ensuring availability of affordable and accurate diagnostic tools to all health facilities and (2) enhancing systems for financing access to diagnostics and antimicrobial medicines. Achieving progress in Sub-Objective 6 requires identification of the microorganisms present in infected wounds using appropriate diagnostic tools and using those results to make evidence-based and contextually relevant clinical and prescribing decisions. The success of this rests fundamentally on high-functioning, multi-disciplinary teams.

We noted (above) how the Call for Funding identified 3 of the 10 Fleming Fund objectives as priorities. This explicitly excluded the Fleming Fund Objective of ‘building laboratory capacity for diagnosis’. Furthermore, the reference to multi-disciplinarity did not refer to laboratory science. However, the outcomes expected from projects did include ‘the use of microbiology data to inform decision-making’. This illustrates both the mixed messaging coming from the Call and the underlying challenge of attempting to isolate specific components of AMR.

Most public hospitals in Uganda lack the capacity (equipment and consumables), human resource and expertise to undertake effective, timely and free microbiology testing. One of the intern doctors interviewed had trained and worked in Mbale Regional Referral Hospital in north-east Uganda (520 km from Fort Portal). This enabled him to draw interesting comparisons between the two facilities in swabbing practices:

The main thing we are doing here is blood culture, swabs and culture and sensitivity testing so at least we are able to prescribe the best antibiotics.

[Was it like that in Mbale?]

No – it wasn’t easy. We did culture and sensitivity testing there but we had to use a private laboratory that charged 30,000 shillings per case^1^. About 50% of people were able and willing to pay for this service. The hospital microbiology laboratory did not function; they lacked consumables, culture medium ….

[Did patients understand the reason for the testing?]

Yes, they did – it was more a question of money.

FPRRH is an exception. The laboratory at FPRRH is supported by the Infectious Disease Institute (IDI) with funding from a range of international partners. As such, it offers excellent laboratory facilities. The IDI team has invested considerably in encouraging health workers in FPRRH to send samples to the laboratory for ‘Culture and Sensitivity Testing’. Relationships with the IDI laboratory pre-dated the MSI. We were aware that IDI had found it very difficult to persuade local health workers to take swabs and samples for testing. A presentation by the IDI team to the IPC Committee in 2018 noted the absence of microbiology testing until 2016. The significant refurbishment of the laboratory in 2018, coupled with capacity-building, improved power supplies and a constant supply of reagents and general supplies created new opportunities. Despite this situation, the presentation concluded that: *There is very limited utilisation of the* *microbiology* *laboratory at FPRRH.*

A subsequent presentation in 2019, and despite significant attempts by the laboratory team to raise awareness on the wards through short training sessions, echoed this concern:

There is low up take of available microbiology diagnostics at FPRRH.

Prior to the commencement of the intervention, most of the patients in FPRRH were prescribed antibiotics on the basis of experiential knowledge (of clinicians). This is influenced by prior education, reference to clinical guidelines and heavily circumscribed by what is available, and perceptions of what is available, in pharmacy stores. This process is termed ‘ empirical therapy’.^2^ There is increasing evidence that many of the most commonly used (and cheaper) antibiotics available in low resource settings (including public hospitals in Uganda) are no longer effective in treating infections; the bacteria have become resistant to them. Continued and extended use of these antibiotics on resistant infections will fail to deliver positive patient outcomes and waste antibiotics, contributing to AMR and hospital costs. Where, as in many RRHs in Uganda, there is no capacity for laboratory testing, doctors have little choice but to throw what is available at patients. The use of microbiology testing in the hospital is beneficial both for individual patient management (ensuring they get the optimal antibiotic for the infection they have) and for creating evidence-based policies to guide empirical antibiotic use.

## Bacteriology-Based (Individualised) Prescribing

The priority for the MSI, in the first instance, was to create the opportunity for individual rational prescribing based on culture and sensitivity testing. From the point of taking a wound swab or blood sample, it can take 5–7 days for antibiotic susceptibility results to be returned to the ward. Taking this into account, alongside the urgency of treating sepsis without delay, many women are prescribed an initial empirical antibiotic regimen until test results are received and communicated to a prescriber. In the Ugandan context, this means waiting for a doctor to reassess the patient and prescription. And it could take many days for the same doctor to review that case.

## Facilitating Culture and Sensitivity Testing

The qualitative interviews with health workers on the PNG ward indicate the priority that they all now ascribe to culture and sensitivity testing. Indeed, it is the normalisation of this process, through substantial and sustained behaviour change, that has underpinned the emergence of highly effective team working . And this, in turn, lies at the heart of intervention success (discussed below). The following midwife describes how this change has happened and the complex chain of events involved:

If we see pus, we do a swab – but those things have just come.

[So, you were not swabbing before?]

We were not taking swabs – because if you took a swab it stayed in the laboratory for a month and there was no one to follow up. Patients stayed with us for over a month and there was no follow up at all. Those people (K4C midwives) do follow up and within 3 days they have results. They bring them to the doctor, so the doctors are ready to work on them and that’s how we have reduced sepsis on the wards. It’s great indeed I’m very happy.

[So, before the project – was it that there was trouble in the laboratory or that no one here was picking the results from the laboratory – was it a communication problem?]

Yes at first we sent the swabs there and it could stay there 2 weeks with no one to follow up the swabs – the laboratory had a problem - they could tell you we don’t have things to use –‘you wait’ – but nowadays things are there to use – you go and they bring back the results when they are out.

[Does having the phone on the ward help?]

Yes, now we use the phone that was bought for us to call the laboratory and also to receive results – it is TOO GOOD! Before we had the phone, we had to move from our ward to the laboratory - if we wanted to speak to the laboratory or take a sample we had to walk up there.

[And did people do that?]

No - some did, and others refused especially when we are alone on duty. So to do all those things – you are tired - you are alone – now [K4C midwife] has come, she can go to the laboratory and they are helping with dressing so for you – you are giving treatment – you are recording in the files – the work now is easy.

[Is it only K4C midwives contacting the laboratory? If they were not there would you do that?]

Yes, these days I’m used to it. We go there – we get the results – we call the doctor – we have the phone - Now we can call a doctor and get immediate follow-up.

[What happens when the results are back?]

We give the results to the doctor – he is the one to decide if they have resistance or not and if they have resistance to some antibiotics they change – if not they continue.

Although she refers to earlier experiences and perceptions of laboratory functionality, there are also issues concerning leadership and roles in terms of taking swabs in the first place and ensuring these physically get to the laboratory without delay. To put this in context, the laboratory is about a 10-minute walk from the ward and often there are only two midwives working on the ward. Leaving critically ill patients for a period of about 30 minutes is a real problem. This is another example of the mundane, minutiae of operational practice on the ward captured through continual observation and dialogue that would be missed using many other research methods and yet indicated a significant systemic weakness and facet of behaviour. The process of taking the swabs to the laboratory has now been streamlined with the laboratory collecting samples after the morning ward round. This also provides an opportunity to engage with the team on the ground. Where necessary, the midwives will physically take swabs to the laboratory during the day. This has been facilitated by the additional human resource provided through the project.

We also found at an early stage in the project that there was a major problem with the communication of results between the laboratory and the ward. The laboratory was attempting to contact the doctor who originally signed the swabbing forms. In practice, the presence of senior doctors on the ward is, at best, sporadic and it is intern doctors who are present for signing. But intern doctors inevitably move around and rotate. Guided by previous experience of an initiative to co-design and implement maternal early warning scores in Mulago Hospital (Ackers and Ackers-Johnson 2016), we were aware that reliance on the personal mobile phones of these doctors and health workers, who receive no funding to cover phone costs, leads to severe communication breakdown. We were concerned too at the impact of having midwives leave the ward perhaps several times a day to physically visit the laboratory for results. On that basis, the project supplied a landline in the nursing station to facilitate smooth and efficient laboratory-ward communication. This initially worked well but on a later visit we found the phone in a cupboard as some staff had been using it for other calls. We later provided fixed housing for the phone on the desk and a book to record calls.^3^

A representative of the laboratory echoes the midwife’s perception of improved behaviour change in swabbing and prescribing practices.

I have seen a very great improvement. Yes, there are bottlenecks, but we have really achieved a lot. Restricting our discussion to post-natal; the utilisation of diagnostics in regard to microbiology previously was very low. The staff didn’t appreciate the value of prescribing on the basis of a laboratory report. Prescribing practices were poor; how often did the doctors come to check patients? They were wasting time. People believed we can use antibiotics the way the Ministry has put them into the clinical guidelines but some of these are not working so they really need to be guided by the bacterial reports. The uptake is really good. The number of samples has increased. Patients are being investigated along the lines of bacteriology – they really endeavour to pick results and the results are directly influencing prescribing behaviour. Although it’s not been documented, the reactions from the nurses give us good news. The patient stay on the ward is actually reducing. We evidenced this last year when we monitored some few patients who had stayed up to 30 days on the ward without getting a bacterial report. From the time they prescribed on the basis of the bacteriology report it took only 7 days for the patient to improve and finish treatment. On the 8^th^ day they were able to close the wound and on the 9^th^ the patient was discharged. This was during the project – last September. We have worked with the K4C team to do CMEs on IPC and swabbing. The main impact has been on rational prescribing – on a scale of 1-10 I’d say we are now at 7.8! It has helped us to inform the clinicians on best practice in terms of prescription and having done that we have reduced patient stay on the ward and then even in terms of costs – shorter stays on the wards and having fewer attendants on the wards.

The impact of the focus on wound care and culture and sensitivity testing is explained by a local midwife. She had taken a particular interest in the use of sugar in wound care prior to the project (in Sudan) and had previously worked alongside K4C staff and British nursing students on the labour ward, so relationships were strong. She describes the impact the project has had on her personally and on the ward and patients. She notes that, prior to the project, empirical prescribing of antibiotics was not effective, and this lack of effectiveness was compounded by prolonged prescribing of the same antibiotics. Importantly, she also specifically recognised the role that clinical pharmacists are now playing:

You came in at a critical time [and] brought new skills. Before there was no culture and sensitivity testing. Some of us knew about it but had never used it – even the doctors. When you came in it is me who benefitted most; I was carrying a very heavy burden and you helped me. You came as a combined team. We have not lost any women from sepsis since the project started. [Ugandan midwife employed by K4C] came. I had worked with her on labour ward with your students. Even the laboratory has started to respond – the burden was lifted, and everyone started getting involved.

We did use culture and sensitivity tests in Mulago (National Referral Hospital) but with not much emphasis and sometimes you have your interests on other things and we left it to the doctors. Here much of the things are now done by nurses/midwives – like doing culture and sensitivity tests. We knew culture and sensitivity would get results. Now I try to do the septic patients first. Before we noticed some were not getting better and we did not pay much attention to how this woman has been on this treatment for so long and you just gave her more antibiotics. […] now [the pharmacist] comes on the ward daily and looks around and helps us as sometimes the intern doctors are busy and lack supervision. Before we used the same medicines – same – same – we just gave what was prescribed.

The Global Point Prevalence Survey completed in May 2019 monitored the use of microbiology testing in prescribing behaviour. At this point, prior to project engagement, no laboratory results were recorded in the files of those patients in the gynaecology or post-natal wards who had been prescribed antibiotics and 100% of antibiotic prescribing was on an empirical basis (Table 5.1).

**Table 5.1:** Antibiotic prescribing based on laboratory results

	Gynaecology (*n* = 22)	Post-natal (*n* = 20)
No. of patients on antibiotics	10	18
Microbiology lab report in notes	0	0

*Source* Results of G-PPS, May 2019 as reported to FPRRH IPC Committee

We have previously noted the remarkable and very rapid improvement in wound management and swabbing once the MSI began to engage. The FPRRH laboratory has excellent documentation systems in place. The results from the laboratory indicate major behaviour change on the ward with 95% of all suspected sepsis cases tested once the project was implemented (Table 5.2).

**Table 5.2:** Volume and proportion of suspected sepsis cases sent for laboratory testing

Time frame	Suspected sepsis cases	Culture and sensitivity tests performed	% Tested
January 1st, 2019–July 8th, 2019	50	0	0
July 9th, 2019–July 21st, 2019	16	3	19
July 22nd, 2019–January 31st, 2020	76	74 (2 had missing data)	95

*Source* FPRRH Laboratory

Laboratory results from these tests were subsequently located in the files of 67 of the 74 (90.5%) patients who had had a swab taken.^4^ Although this emphasises the need to further improve record-keeping, this level of documentation represents a remarkable achievement in the context.

## Utilising Laboratory Data and Antibiograms to Improve Empirical Therapy

In addition to providing the opportunity for an individualised treatment regime, the submission of samples to the laboratory builds up a wider evidence base of resistance patterns specific to that hospital setting. This evidence base enables microbiologists to identify more general resistance patterns that determine the expected efficacy of individual antibiotics. The collection of a sufficiently robust evidence base enables the hospital to generate what is known as an ‘ antibiogram’. An antibiogram is a collection of data, based on laboratory testing of the pathogens in a specific facility that summarises patterns of resistance to different antimicrobial agents (or antibiotics). Although international and national trends in resistance patterns can be identified, regional and facility-specific patterns enable even closer targeting of antibiotics. The intern doctor who had moved from Mbale hospital (above) reported his perception of regional differences in resistance patterns:

The challenge here is that bacteria are more resistant than in Mbale. Here bacteria are often resistant to more than one antibiotic. We have been culturing E.coli here and there is much resistance.

Where a hospital antibiogram exists, initial prophylactic and empirical prescribing can be informed by local evidence and has a much higher chance of success. The presence of an antibiogram with associated awareness raising and sensitisation amongst all staff and especially medical interns would have major impacts on empirical prescribing across the hospital. Not only can it help reduce the reliance on and overuse of specific antibiotics, a driver of resistance, it can act as a guide for procurement of more potent antibiotics at the hospital. The MSI has played an important part in creating the evidence base for a hospital antibiogram. Prior to the MSI, FPRRH did not have the volume of laboratory results to create the necessary evidence base for a hospital antibiogram. A member of the pharmacy team describes how this has changed:

If you go to maternity, you will notice a very big change. The ward sends the biggest volume of swabs now to the laboratory because those people [midwives] are aware.

The laboratory scientist confirms this:

On the basis of the increased swabbing we hope to be in a position to have an antibiogram. This will be very informative. The sample size is now very adequate. The antibiogram will be good for the clinicians to guide prescribing and it will be good for the patients.

He goes on to refer to the contribution that swabbing has made to an understanding of health care acquired infection and IPC monitoring. That week had seen 3 cases of *Acinetobacter*, a multidrug-resistant organism on PNG ward:

Having the bacteriology reports coming through has also helped us. We want to share a report that will be coming next week on the trends of pathogens being identified and sensitivity patterns to see if this is the same organism from one patient to another so we can have targeted interventions when it comes to IPC. We can also see if it is airborne or a contagious organism and then we can improve on the IPC. Now we don’t have that good background on IPC. It has helped us to do localised epidemiology on hospital acquired infections.

Not only has the practice of sending samples to the laboratory improved dramatically over the period of the project, the laboratory reports that PNG is responsible for nearly all of the laboratory testing that is taking place in the hospital and contributing to the forthcoming antibiogram. The specific impact of team working with IDI has underpinned all of these developments:

[Have you noticed more swabs being taken from other wards?]

Actually no – it is mainly post-natal and surgical (IDI has had a project on surgical). IDI cover the whole hospital, but we have had some real challenges persuading people of the value of prescribing on the basis of laboratory results. Some people don’t have the time to really investigate these patients – some base prescribing on clinical guidelines. OK, but they see what is in those guidelines is no longer working. We are coming up with a highly resistant Acinetobacter and we should not be prescribing certain antibiotics – all these forces will come if we have the right information.

In the time since this interview took place, an operational antibiogram has been produced by laboratory staff at FPRRH, shown in Table 5.11.

This data set, drawn from the laboratory, includes wound swabs and other samples illustrating the more extensive growth in sampling on the ward. Due to laboratory capacity constraints commonly seen in lower-income countries, samples are only grown and tested aerobically, meaning the possible presence of any anaerobic bacteria^5^ is missed. Of the 116 total samples sent to the laboratory, 63 (54%) reported positive growth^6^ and 51 (44%) reported no growth. Only 2 samples had probable contamination highlighting the success of laboratory protocols (Table 5.3).

**Table 5.3:** Summarised data for microbiology testing on samples drawn from post-natal and gynae wards, FPRRH (July 2019–January 2020)

Sample type	No. Samples	Growth	No growth	Contamination
Pus swabs/Aspirates	82	52	28	2
High vaginal swabs	16	10	6	0
Blood	16	1	15	0
CSF	1	0	1	0
Urine	1	0	1	0
Total	116	63	51	2

*Source* FPRRH/IDI Laboratory

A single sample may be attributed to a single bacterial isolate, or less commonly, multiple bacterial isolates indicating a polymicrobial infection. This explains the higher number of isolates reported in Tables 5.4, 5.5, 5.6, and 5.7 relative to Table 5.3. Culture identification (Table 5.4) and antibiotic sensitivity testing were performed for all positive isolates^7^ grown. The most common organism isolated was *Escherichia coli* (28%), followed by *Acinetobacter species* (18%) and *Staphylococcus aureus* (16%). This corresponds with a study of 314 surgical site infections at Mulago National Referral Hospital, where 23.7% of isolates were *E. coli*, 21.1% were *S. aureus* and 17.1% were *Acinetobacter species* (Seni et al. 2013). The resistance patterns of these bacteria are further explored in Tables 5.4, 5.5, 5.6, and 5.7.

**Table 5.4:** Number of isolates of each bacterial species identified from the samples tested

Isolate ID	No. of isolates
*Escherichia coli*	26
*Acinetobacter species*	17
*Staphylococcus aureus*	15
*Klebsiella species*	13
*Enterococcus species*	7
*Coagulase Negative Staph*	4
*Proteus mirabilis*	4
*Streptococcus pyogenes*	2
*Streptococcus agalactia*	1
*Pseudomonas aeruginosa*	1
*Providencia stuartii*	1
*Candida species*	1
*Raoultella ornithinolyticus*	1
Total isolates	93

**Table 5.5:** Antibiotic resistance patterns of *Acinetobacter species* isolated from PNG ward

Acinetobacter species (*n* = 17)				
Antibiotic agent	Susceptible	Intermediate	Resistant	Resistant (%)
Imipenem	1	1	15	88
Cefepime	0	1	16	94
Cefotaxime	0	1	15	88
Trimethoprim/sulfame	2	5	10	59
Doxycycline	11	1	5	29
Ciprofloxacin	2	0	15	88
Amikacin (*n* = 9)	8	0	1	11
Piperacillin/Tazobactam (*n* = 9)	0	1	8	89
Gentamicin	1	0	16	94

*Source* FPRRH Laboratory

**Table 5.6:** Antibiotic resistance patterns of *Escherichia coli* isolated from PNG ward

Escherichia coli. (*n* = 26)				
Antibiotic agent	Susceptible	Intermediate	Resistant	Resistant (%)
Imipenem (*n* = 25)	25	0	0	0
Gentamicin	19	1	6	23
Chloramphenicol	22	1	3	11
Ampicillin	2	0	24	92
Cefotaxime	5	1	20	77
Ciprofloxacin	17	1	8	31
Doxycycline (*n* = 23)	2	3	18	78
Piperacillin/Tazobactam (*n* = 19)	16	1	2	11
Trimethoprim/sulfame (*n* = 25)	2	0	23	92
Cefuroxime	4	1	21	81

*Source* FPRRH Laboratory

**Table 5.7:** Antibiotic resistance patterns of *Staphylococcus aureus* isolated from PNG ward

Staphylococcus aureus (*n* = 15)				
Antibiotic agent	Susceptible	Intermediate	Resistant	Resistant (%)
Chloramphenicol	14	0	1	7
Gentamicin	14	0	1	7
Cefoxitin (*n* = 13)	11	0	2	15
Trimethoprim/sulfame	9	1	5	33
Ciprofloxacin (*n* = 14)	11	0	3	21
Clindamycin	15	0	0	0
Erythromycin (*n* = 13)	6	1	6	46

*Source* FPRRH Laboratory

The antibiotic susceptibility of isolates was tested utilising the disk diffusion technique against a specific panel of antibiotics to best determine its resistance potential. The two antibiotics most commonly prescribed in the PNG ward: ceftriaxone and metronidazole, are not specifically mentioned in the tables below. These antibiotics are often prescribed together prophylactically prior to any laboratory testing. Metronidazole is commonly used to treat anaerobic bacteria (Smith 2018; Shafquat et al. 2019) and as such testing for this antibiotic requires laboratories capable of simulating anaerobic conditions. This is not possible at FPRRH. Ceftriaxone, on the other hand, is a member of the cephalosporin group of antibiotics. In this case, other members of the same family (such as cefepime or cefotaxime) with similar mechanisms of action can be used to infer resistance.

Table 5.5 evidences an alarmingly high level of resistance across all antibiotics tested against the 17 *Acinetobacter* isolates with the exception of doxycycline and amikacin (5 and 1 resistant isolates, respectively). That there were no isolates susceptible to cefepime or cefotaxime, fourth- and third-generation cephalosporins, respectively, is cause for concern. Not only are these some of the most recent iterations of antibiotics, but the fact that the primary antibiotic of choice on the ward is ceftriaxone shows *Acinetobacter* infections could leave people vulnerable. Equally, once an infection is present, few other antibiotics are shown to be effective.

The most prevalent bacterial species identified from the tests was *E. coli.* Table 5.6 shows that *E. coli* displays mixed levels of resistance, leaning towards being either highly susceptible or highly resistant depending on the antibiotic of choice. Again, there is high resistance to cefotaxime (20/26) as well as cefuroxime (21/26), a second-generation cephalosporin, which adds to the concern that ceftriaxone is losing its effectiveness. Thankfully, imipenem^8^ has been shown to be 100% effective against the isolates tested, promoting its use as a secondary option.

The third most prevalent isolate was *S. aureus*. (Table 5.7) which showed minimal resistance to all antibiotics with the exception of erythromycin and trimethoprim/sulfame. Additionally, clindamycin was shown to be 100% effective against the isolates tested. Of particular note is cefoxitin which performed strongly with 11 susceptible and 2 resistant isolates. Cefoxitin is a second-generation cephalosporin and acts as an indicator for MRSA (methicillin-resistant *S. aureus*), a key metric for AMR.

## Utilising Local *Staphylococcus aureus* Populations as a Case Study to Investigate Trends in Resistance Patterns

Carried by roughly 30% of the human population, *Staphylococcus aureus* is a Gram-positive coccus found frequently in the nasopharynx, respiratory tract and on skin. Whilst commonly found as a commensal^9^ organism, *S. aureus* is also a major human pathogen with the ability to cause a wide range of clinical infections resulting in skin (where the skin has been broken, for example, from a wound or surgery) and respiratory diseases (Tong et al. 2015). Infections occur most regularly in hospitalised patients where the consequences can be severe. Though often easily treated with antibiotics, if left neglected the infection can worsen and spread. *S. aureus* is one of several organisms commonly linked to bloodstream infections and cases of sepsis. A large meta-analysis of community acquired bloodstream infections in Africa examined 5578 patients with non-malaria bloodstream infections, where 531 (9.5%) cases were due to *S. aureus* (Reddy et al. 2010). That being said, treatment has become increasingly complicated due to the rise of methicillin-resistant *S. aureus* (MRSA) which is now often multi-resistant. As such, methods of infection prevention are becoming more valuable (Kluytmans et al. 1997).

With a better understanding of where and how resistance arises, steps can be taken to prevent it. Ongoing research forming part of Ackers-Johnson’s microbiology doctorate (Ackers-Johnson 2020) investigates in depth the mechanisms of antibiotic resistance and their respective relation to different strains of *S. aureus*. As part of this, *S. aureus* isolates obtained clinically from across all wards at FPRRH were investigated (Table 5.8), as well as isolates obtained from the hands of healthy members of the public (Table 5.9)—all within the hospital catchment area.

**Table 5.8:** Antibiotic resistance patterns of *Staphylococcus aureus* from all wards at FPRRH

Staphylococcus aureus (*n* = 70)				
Antibiotic agent	Susceptible	Intermediate	Resistant	Resistant (%)
Chloramphenicol (*n* = 59)	53	0	6	10
Gentamicin (*n* = 68)	54	2	12	18
Cefoxitin (*n* = 21)	13	0	8	38
Trimethoprim/sulfame (*n* = 57)	25	6	26	46
Ciprofloxacin (*n* = 69)	51	4	14	20
Clindamycin (*n* = 62)	62	0	0	0
Erythromycin (*n* = 69)	47	1	21	30
Tetracycline (*n* = 53)	24	0	29	55

*Source* These bacteria were clinically isolated from samples across all wards at FPRRH between August 2017 and April 2019. Whilst 70 isolates were assessed, not all could be tested against all antibiotics due to supply constraints as the laboratory was in its initial stages of increasing capacity

**Table 5.9:** Antibiotic resistance patterns of *Staphylococcus aureus* isolated from hand swabs of the general public

Staphylococcus aureus (*n* = 125)				
Antibiotic agent	Susceptible	Intermediate	Resistant	Resistant (%)
Chloramphenicol (*n* = 124)	87	0	37	30
Gentamicin (*n* = 123)	116	0	7	6
Cefoxitin	98	0	27	22
Trimethoprim/sulfame	54	4	67	54
Ciprofloxacin	81	0	44	35
Clindamycin (*n* = 124)	24	53	47	38
Erythromycin (*n* = 124)	39	28	57	46
Tetracycline	57	31	37	30

*Source* Ackers-Johnson (2020)

As previously noted, production of an accurate antibiogram requires a large number of samples. Whilst the antibiotics’ effectiveness relative to each other remains largely similar, a greater percentage of resistant isolates can be seen when assessing the 70 samples acquired from across the hospital (Table 5.8) compared to the 15 samples from the PNG ward (Table 5.7). In the case of gentamicin and cefoxitin, the rates of resistance are more than doubled. Interestingly, even when observing the larger sample size, clindamycin has still been shown to be 100% effective.

Observing the resistance profiles of *S. aureus* found on the hands of healthy members of the public within the catchment area of FPRRH highlights how AMR is not a problem reserved exclusively to hospitalised patients. Table 5.9 shows relatively high levels of resistance across all antibiotics tested with the exception of gentamicin at 6%. Moreover, clindamycin, which was shown to be 100% effective against isolates obtained clinically at FPRRH, has much higher levels of resistance within the local community at 38%. With such levels of resistance present, the requirement for effective IPC is further emphasised to reduce the incidence of infection from harmful resistant bacteria already present on skin. This raises questions about the role that attendants may play as vectors of infection on the wards especially when they are involved in clinical roles (wound and canula management) and the impact of stock-outs of iodine on surgical safety practices in theatre.^10^ Hsieh et al. (2014) generated a theoretical model to predict the impact of hospital visitors on nosocomial transmission and spread to the community concluding that, ‘transmission rates of infective residents in the community and of infective visitors at the healthcare facility have a decisive impact on disease eradication/persistence’ (2014: 20). These concerns are emerging in current research on COVID-19 (Halbfinger 2020; Hsu et al. 2020; Wee et al. 2020).

Comparing the situation at FPRRH to that of the National Referral Hospital (Mulago Hospital, Table 5.10), the results again seem relatively similar with trimethoprim/sulfame exhibiting exceedingly high levels of resistance, with gentamicin and chloramphenicol being amongst the strongest performing antibiotics. Conversely, of particular note is clindamycin. 40.6% of *S. aureus* isolated from surgical site infections at Mulago hospital displayed resistance to clindamycin, similar to that of the community hand swabs in Fort Portal (38%), whilst clinical isolates from FPRRH were 100% susceptible. Such data emphasises the need for further advanced research.

**Table 5.10:** Antibiotic resistance patterns of *Staphylococcus aureus* clinically isolated from surgical site infections at Mulago Hospital

Staphylococcus aureus (*n* = 64)	
Antibiotic agent	Resistant (%)
Chloramphenicol	15.6
Gentamicin	18.8
Ampicillin	100
Trimethoprim/sulfame	89.1
Ciprofloxacin	29.7
Clindamycin	40.6
Erythromycin	46.9
Tetracycline	42.2

*Source* Seni et al. (2013)

Tables 5.5, 5.6, 5.7, 5.8, and 5.9 highlight the necessity of microbiology testing in controlling infection and promoting antimicrobial stewardship. It is clear from the data that there is no antibiotic ‘silver bullet’ available in this local setting capable of treating an infection of unknown identity. All the bacteria assessed at FPRRH had some level of resistance to all antibiotics tested, except for imipenem (100% effective against *E. coli*) and clindamycin (100% effective against *S. aureus*). As such, prescribing without the respective laboratory data could well prove to be in vain. The unchecked overuse of these ineffective antibiotics not only compromises patient safety and acts as an economical burden, but also further drives the development of resistance—with increased levels of antibiotics in the local environment it is possible that other bacteria unrelated directly to the cause of infection can progress to acquire resistance causing issues in the longer term. The construction of the antibiogram (Table 5.11) will go a long way to address these problems, the reliability of which itself has been greatly aided by the increased volume of samples being taken as a by-product of effective patient care.

**Table 5.11:**
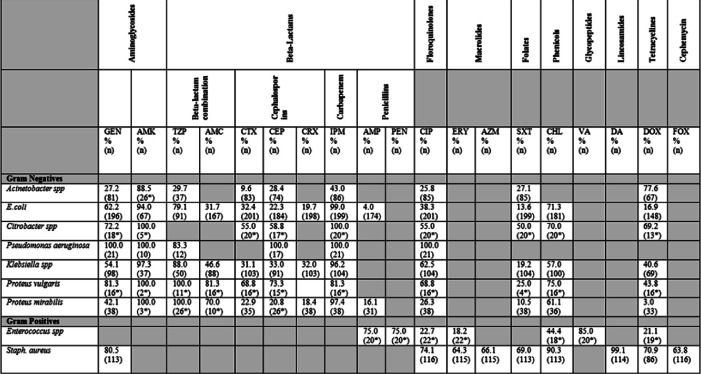
Hospital Antibiogram (July 2020) showing percentage susceptibility of isolates based on data collected from January 2019 to June 2020 at Fort Portal Regional Referral Hospital

**GEN**-Gentamycin, **AMK**-Amikacin, **TZP**-Piperacillin/Tazobactam, **AMC**-Amoxicillin/Clavulanate, **CTX**-Cefotaxime, **CEP**-Cefepime, **CRX**-Cefuroxime, **IPM**-Imipenem, **AMP**-Ampicillin, **PEN**-Penicillin, **CIP**-Ciprofloxacin, **ERY**-Erythromycin, **AZM**-Azithromycin, **SXT**-Sulphamexazole/Trimethoprim, **CHL**-Chloramphenicol, **VA**-Vancomycin, **DA**-Clindamycin, **DOX**-Doxycycline, FOX-Cefoxitin **(n)**-Number of isolates, **%**-Percentage of susceptibility, *Results based on fewer than 30 isolates are less reliable and should be interpreted with caution

 The antibiogram displays the percentage susceptibility of isolates recovered from microbiological samples analysed at FPRRH. The first column indicates the organisms that were assessed, with the remaining columns indicating the antibiotics that they were tested against, the percentage of isolates that were susceptible to the antibiotic, and (n)—the total number of isolates tested. It is worth noting that data where fewer than 30 isolates have been assessed are less reliable and hence any conclusions/prescribing should be done with caution. These have been marked with an asterisk (*) and highlighted yellow.

As previously mentioned, a wide range of resistances to various antibiotics can be seen across the data. As a general rule, if an antibiotic has been shown to be less than 80% effective against a particular organism, then it should not be prescribed as an empirical therapy for serious infections. As per the antibiogram, a number of antibiotics fall short of this benchmark. With relatively more expensive and less commonly prescribed antibiotics (e.g. Amikacin and Imipenem) often outperforming those that are frequently used, it is important that the antibiogram plays a role in the hospitals’ antibiotic procurement plan as particular treatments are simply not health or cost-effective. Whilst budget constraints may not be able to permit the frequent use of such tailored antibiotics, clear policies and guidelines should be put in place by the MTC to govern their usage in the care of critical patients.

Whilst there is a definite value in utilising an antibiogram, it is also useful to point out that it has limitations. Being used for empirical diagnoses, there are no guarantees that the antibiotic of choice will be effective even if it has a very high theoretical rate of success. This could be due to the performance of the antibiotic (and antibiotic quality) or the (mis)identification of the causal bacteria. As such, the presence of an antibiogram does not remove the essential need for clinicians to utilise the laboratory services available to provide definitive information in conjunction with patient observations to deliver optimal treatment.

The growth in wound management coupled with increased laboratory testing has created task-shifting opportunities (and workloads) for nurses and midwives as staff providing continuity of care on the wards. It has also created new opportunities for the engagement of clinical pharmacy. Chapter 10.1007/978-3-030-62662-4_6 discusses these issues in more depth and then assesses one of the major, structural, barriers to further improvement: supply chain dynamics in Ugandan public hospitals.

## Footnotes

1 About £6.

2 Empirical therapy refers to, ‘Treatment given without knowledge of the cause or nature of the disorder and based on experience rather than logic. Sometimes urgency dictates empirical treatment, as when a dangerous infection by an unknown organism is treated with a broad-spectrum antibiotic while the results of bacterial culture and other tests are awaited’ https://medical-dictionary.thefreedictionary.com/empirical+treatment.

3 The lack of land lines in Ugandan health facilities is a major factor inhibiting communication both with patients and other health facilities.

4 For four patients, the results had gone missing from the file; for two patients, the test was not completed because the IDI laboratory was closed over Christmas and New Year and one patient’s lab test was not completed because the patient had discharged herself against medical advice.

5 Bacteria that thrive in the absence or minimal presence of oxygen.

6 Indicating that bacteria are present.

7 The term isolate refers to the isolation of a particular bacterial colony from amongst others on the agar plate.

8 We note the increased ordering of meropenem in the hospital procurement plan in Table 10.1007/978-3-030-62662-4_6.

9 Can live naturally on human skin without causing harm.

10 At one point during stock-outs, staff had only saline to use.
